# The effect of clodronate and antioestrogens on bone loss associated with oestrogen withdrawal in postmenopausal women with breast cancer

**DOI:** 10.1054/bjoc.2001.1729

**Published:** 2001-04

**Authors:** T Saarto, L Vehmanen, I Elomaa, M Välimäki, P Mäkelä, C Blomqvist

**Affiliations:** Department of Oncology, Helsinki University Central Hospital, PO Box 180, FIN-00029, HUCH, Finland; Division of Endocrinology, Department of Medicine, Helsinki University Hospital, PO Box 340, FIN-00029, HUCH, Finland; Department of Diagnostic Radiology, Helsinki University Hospital, PO Box 180, HUCH, FIN-00029, Finland; Department of Oncology, Uppsala University, Sweden and Department of Oncology, Helsinki University Central Hospital, PO Box 180, HUCH, FIN-00029, Finland

**Keywords:** antioestrogens, bone mineral density, bisphosphonates, breast neoplasms, postmenopausal osteoporosis, toremifene

## Abstract

In this study we report bone mineral density (BMD) changes during clodronate and antioestrogen treatment in women with breast cancer having discontinued hormone replacement therapy (HRT) at the time of operation compared to women who had not used HRT immediately before the operation. 61 postmenopausal women with operable breast cancer were treated with the adjuvant antioestrogen tamoxifen 20 mg or toremifene 60 mg daily for 3 years. All patients were randomized to clodronate (1.6 g daily orally) or control groups for 3 years. 23 patients had recently (recent users) and 38 never or not for at least 1 year before operation used HRT (non-users). BMD of lumbar spine and femoral neck were measured before antiresorptive therapy (antioestrogens and clodronate) and at 1, 2, 3 and 5 years thereafter. All patients were disease-free at the time of BMD measurements. Patients who had recently used HRT had more significant bone loss as compared to HRT non-users at 3 years in lumbar spine – 3.0% vs. + 1.2% (*P*< 0.001), but not in femoral neck – 0.4% vs. + 1.7% (*P* = 0.27). Adding 3-year clodronate treatment to antioestrogen therapy improved BMD marginally at 3 years: lumbar spine + 1.0% vs. –1.7% (*P* = 0.01) and femoral neck + 2.4% vs. –0.4% (*P* = 0.12). This was also seen at 5 years of follow-up, 2 years after termination of the antiresorptive therapy: HRT recent users vs. HRT non-users in lumbar spine –6.5% vs. +0.5% (*P*< 0.0001) and in femoral neck –4.8% vs. –1.5% (*P* = 0.38); and clodronate vs. controls in lumbar spine –1.0% vs. –3.2% (*P* = 0.06) and in femoral neck –0.1% vs. –5.2% (*P* = 0.001, respectively). The type of endocrine therapy (tamoxifen and toremifene) had no significant influence on BMD changes. We conclude from this study that postmenopausal women who have recently discontinued HRT experience more rapid bone loss than HRT non-users. Neither 3-year antioestrogen therapy alone nor antioestrogen together with clodronate could totally prevent the bone loss related to HRT withdrawal in lumbar spine, even though clodronate seemed to retard it. © 2001 Cancer Research Campaignhttp://www.bjcancer.com


British Journal of Cancer (2001) 84(8), 1047–1051
© 2001 Cancer Research Campaign
doi: 10.1054/ bjoc.2001.1729, available online at http://www.idealibrary.com on IDEAL®

http://www.bjcancer.com

### The effect of clodronate and antioestrogens on bone loss associated with oestrogen withdrawal in postmenopausal women with breast cancer

**T Saarto[1], L Vehmanen[1], I Elomaa[1], M Välimäki[2], P Mäkelä[3] and C Blomqvist[4]**

[1]Department of Oncology, Helsinki University Central Hospital, PO Box 180, FIN-00029, HUCH, Finland; [2]Division of Endocrinology, Department of Medicine, Helsinki University Hospital, PO Box 340, FIN-00029, HUCH, Finland; [3]Department of Diagnostic Radiology, Helsinki University Hospital, PO Box 180, FIN-00029, HUCH, Finland; [4]Department of Oncology, Uppsala University, Sweden and Department of Oncology, Helsinki University Central Hospital, PO Box 180, FIN-00029, HUCH, Finland

**Summary** In this study we report bone mineral density (BMD) changes during clodronate and antioestrogen treatment in women with breast cancer having discontinued hormone replacement therapy (HRT) at the time of operation compared to women who had not used HRT immediately before the operation. 61 postmenopausal women with operable breast cancer were treated with the adjuvant antioestrogen tamoxifen 20 mg or toremifene 60 mg daily for 3 years. All patients were randomized to clodronate (1.6 g daily orally) or control groups for 3 years. 23 patients had recently (recent users) and 38 never or not for at least 1 year before operation used HRT (non-users). BMD of lumbar spine and femoral neck were measured before antiresorptive therapy (antioestrogens and clodronate) and at 1, 2, 3 and 5 years thereafter. All patients were disease-free at the time of BMD measurements. Patients who had recently used HRT had more significant bone loss as compared to HRT non-users at 3 years in lumbar spine – 3.0% vs. + 1.2% ($P < 0.001$), but not in femoral neck – 0.4% vs. + 1.7% ($P = 0.27$). Adding 3-year clodronate treatment to antioestrogen therapy improved BMD marginally at 3 years: lumbar spine + 1.0% vs. –1.7% ($P = 0.01$) and femoral neck + 2.4% vs. –0.4% ($P = 0.12$). This was also seen at 5 years of follow-up, 2 years after termination of the antiresorptive therapy: HRT recent users vs. HRT non-users in lumbar spine –6.5% vs. +0.5% ($P < 0.0001$) and in femoral neck –4.8% vs. –1.5% ($P = 0.38$); and clodronate vs. controls in lumbar spine –1.0% vs. –3.2% ($P = 0.06$) and in femoral neck –0.1% vs. –5.2% ($P = 0.001$, respectively). The type of endocrine therapy (tamoxifen and toremifene) had no significant influence on BMD changes. We conclude from this study that postmenopausal women who have recently discontinued HRT experience more rapid bone loss than HRT non-users. Neither 3-year antioestrogen therapy alone nor antioestrogen together with clodronate could totally prevent the bone loss related to HRT withdrawal in lumbar spine, even though clodronate seemed to retard it. © 2001 Cancer Research Campaign http://www.bjcancer.com

**Keywords**: antioestrogens; bone mineral density; bisphosphonates; breast neoplasms; postmenopausal osteoporosis; toremifene

Bisphosphonates are bone-specific drugs, which inhibit bone resorption by inhibiting osteoclast activity (Fleisch, 1995). Bisphosphonates significantly prevent bone loss and osteoporotic fractures in postmenopausal women (Storm et al, 1990; Watts et al, 1990; Harris et al, 1993; Liberman et al, 1995; Black et al, 1996; Karpf et al, 1997; Cummings et al, 1998; Hosking et al, 1998). Tamoxifen has an oestrogen-agonistic effect on bone and therefore also prevents bone loss in postmenopausal women (Love et al, 1992; Ward et al, 1993; Kristensen et al, 1994; Grey et al, 1995; Powles et al, 1996). Women with breast cancer are usually recommended to discontinue previous HRT due to the fear of increasing the risk of breast cancer recurrence. In healthy postmenopausal women bone loss increase after withdrawal of HRT (Lindsay et al, 1978; Christiansen et al, 1981). No data are available as to whether antioestrogen therapy or bisphosphonates are effective enough to inhibit the accelerated bone loss occurring after withdrawal of HRT.

*Received 23 August 2000*
*Revised 6 December 2000*
*Accepted 22 January 2001*

*Correspondence to:* I Elomaa

Toremifene is a close analogue to tamoxifen with demonstrated efficacy in advanced breast cancer (Valavaara et al, 1988). There are 2 studies available on the effect of toremifene on bone mineral density. In our previous report of 2 year antioestrogen therapy the 2 antioestrogens, tamoxifen or toremifene, similarly prevented the bone loss (Saarto et al, 1997b). In another study with a lower dose of toremifen tamoxifen was superior to toremifene (Marttunen et al, 1998).

We have earlier demonstrated in a prospective, open randomized study, that adding clodronate treatment to adjuvant antioestrogen therapy significantly improved BMD in postmenopausal women (Saarto et al, 1997b). We here present (1) the 5-year follow-up results of this study and (2) the impact of previous HRT on bone loss.

### MATERIAL AND METHODS

### Patients

The study population consists of 61 postmenopausal patients with primary operable breast cancer and histologically proven axillary metastases. Patients were treated between March 1991 and July 1993 at Helsinki University Hospital, Department of Oncology. Exclusion criteria included the following: (1) age above 75 years;

(2) a Karnofsky performance index below 70%; (3) other malignancies; (4) breast cancer recurrence at the time of BMD measurements or skeletal metastases within 6 months after the measurement of BMD; (5) previous diseases or medications having an influence on bone metabolism.

Informed consent was obtained from all participants. The study was approved by the Local Ethical Committee, at the Department of Oncology, Helsinki University Hospital. Of the 121 eligible patients, data from 60 patients were excluded from the analyses: 33 due to death, 11 due to breast cancer recurrence, 3 due to protocol violation (patients treated with chemotherapy), 1 patient due to loss from follow-up, 7 because of diseases or medications affecting calcium and bone metabolism and 5 because of missing data of previous HRT. Thus, 61 patients were eligible for analyses. 3 patients interrupted clodronate treatment after a median of 16 months and 2 patients had dose reduction. 2 patients interrupted tamoxifen therapy after a median of 14 months. All these patients are included in the analyses.

23 women had used HRT until the breast operation (recent users), 38 patients had not used HRT (non-users), either never (27 patients) or discontinued at least 1 year before the study entry (11 patients). The mean duration of menopause was 10 years in HRT non-users (from 0.5 to 27 years). Pretreatment characteristics of the subjects in the HRT recent users and non-users, and in the clodronate and control groups are given in Table 1. Women who had not used HRT were significantly older and heavier than HRT recent users ($P$ = 0.013 and 0.001, respectively).

### Treatment and follow-up

All patients underwent surgery with axillary evacuation and total mastectomy or breast-conserving resection and postoperative radiotherapy. Patients were randomly allocated to receive adjuvant antioestrogen therapy: tamoxifen 20 mg or toremifene 60 mg per day for 3 years. In addition all patients were randomized to oral clodronate (Bonefos®, Leiras) 800 mg twice daily for 3 years or controls.

Staging investigations for breast cancer included clinical examination, liver ultrasound, chest X-ray and bone scintigraphy. Basic laboratory tests before randomization included a complete blood count and sedimentation rate, liver enzymes (transaminase, alkaline phosphatase, 5-nucleotidase), serum creatinine, calcium and electrolytes. Patients were interviewed regarding menopausal status, medications, and other diseases before randomization and at 1, 2, 3 and 5 years thereafter. Bone scintigraphy, and determinations of plasma FSH, LH and oestradiol were performed before treatment and at 1, 2, 3 and 5 years. Plasma concentrations of FSH and LH were measured by immunofluorometric assays (IFMA, Wallac, Turku, Finland) and plasma oestradiol levels were measured by a radioimmunoassay (RIA, Farmos, Oulunsalo, Finland). Clinical investigation and basic laboratory tests were repeated every 4 to 6 months with a radiological examination if necessary. The minimum follow-up time was 5 years in all patients.

### Bone densitometry

BMD (g/cm$^2$) was measured by DXA using a Hologic QDR-1000 densitometer (Hologic, Inc, Waltham, MA). BMD was measured at the lumbar vertebrae (L1–L4) and femoral neck in the right femoral area before initiation of therapy and at 1, 2, 3 and 5 years thereafter. The coefficients of variation for precision of the BMD measurments in the lumbar vertebrae and femoral neck were 0.9% and 1.2%, respectively.

### Statistical methods

The influence of different parameters on the changes from start to those of 5-year follow-up of BMD were tested by multivariate regression analyses with backward, stepping elimination of nonsignificant variables: age, weight, clodronate treatment, previous HRT and the type of endocrine therapy (tamoxifen and

**Table 1** Pretreatment characteristics (mean and (SD), median and range, or absolute number and percentage) of patients in clodronate and control groups, and in hormone replacement therapy recent users and non-users

| | Control | | Clodronate | | *P* value | Non-users | | Recent users | | *P* value |
|---|---|---|---|---|---|---|---|---|---|---|
| Number of patients | 32 | | 29 | | | 38 | | 23 | | |
| Age (y) | 61 | (7) | 58 | (7) | NS | 62 | (7) | 57 | (7) | 0.013 |
| Antoestrogen: | | | | | | | | | | |
| Tamoxifen | 16 | 50% | 16 | 55% | NS | 19 | 50% | 13 | 56% | NS |
| Toremifene | 16 | 50% | 13 | 45% | | 19 | 50% | 10 | 44% | |
| Study treatment: | | | | | | | | | | |
| Controls | | | | | | 18 | 47% | 14 | 61% | NS |
| Clodronate | | | | | | 20 | 53% | 9 | 39% | |
| HRT: | | | | | | | | | | |
| non-users | 18 | 56% | 20 | 69% | NS | | | | | NS |
| recent users | 14 | 44% | 9 | 31% | | | | | | |
| Weight (kg) | 70 | (12) | 68 | (8) | NS | 72 | (10) | 65 | (8) | 0.001 |
| Height (cm) | 163 | (5) | 165 | (5) | NS | 164 | (5) | 163 | (4) | NS |
| FSH (U l$^{-1}$) | 62 | 15–109 | 51 | 2–100 | NS | 51 | 2–94 | 62 | 17–109 | NS |
| LH (U l$^{-1}$) | 34 | 14–94 | 35 | 1–89 | NS | 30 | 1–62 | 48 | 12–94 | 0.006 |
| Oestradiol (nmol l$^{-1}$) | 0.02 | 0.02–0.28 | 0.02 | 0.01–0.66 | NS | 0.03 | 0.01–0.66 | 0.02 | 0.02–0.18 | NS |
| Lumbar spine BMD (g/cm$^2$) | 0.970 | (0.149) | 0.950 | (0.139) | NS | 0.950 | (0.144) | 0.975 | (0.143) | NS |
| Normal BMD (≥ 0.960 g/cm$^2$) | 11 | 34% | 10 | 34% | | 13 | 34% | 9 | 39% | |
| Osteopenia (0.790–0.959 g/cm$^2$) | 19 | 60% | 17 | 59% | | 22 | 58% | 13 | 57% | |
| Osteoporosis (< 0.790 g/cm$^2$) | 2 | 6% | 2 | 7% | | 3 | 8% | 1 | 4% | |
| Femoral neck BMD (g/cm$^2$) | 0.783 | (0.134) | 0.768 | (0.119) | NS | 0.771 | (0.136) | 0.775 | (0.111) | NS |

 *© 2001 Cancer Research Campaign*

toremifene). The effect of the independent factors were thereafter tested by a repeated measures ANOVA model using the programs SPSS for Macintosh, with change from baseline BMD as the dependent variable, and with clodronate, HRT and age (above or below median) as grouping variables. No significant interactions between the prognostic factors were found. BMD values are expressed as a percentage of the baseline value. Nonadjusted percentage changes in BMD are shown in the text and tables, and adjusted changes in the figures. Ninety-five percent confidence intervals were calculated for the main nonadjusted outcome measures. Other comparisons were accomplished using the Mann–Whitney test, Chi-Square test or the Wilcoxon matched pair test.

### RESULTS

### Regression analyses

Age ($P$ = 0.001), clodronate treatment ($P$ = 0.005) and previous HRT ($P$ = 0.03) were independent prognostic factor of BMD changes in lumbar spine during the 3-year treatment period, but none in changes of femoral neck. During the whole 5 years follow-up age ($P$ < 0.00001), previous HRT ($P$ = 0.004) and clodronate treatment ($P$ = 0.03) were independent prognostic factor for BMD changes in lumbar spine; and clodronate ($P$ = 0.0008) and age ($P$ = 0.01) for femoral neck BMD. The type of endocrine therapy (tamoxifen and toremifene) or weight had no significant effect on BMD changes.

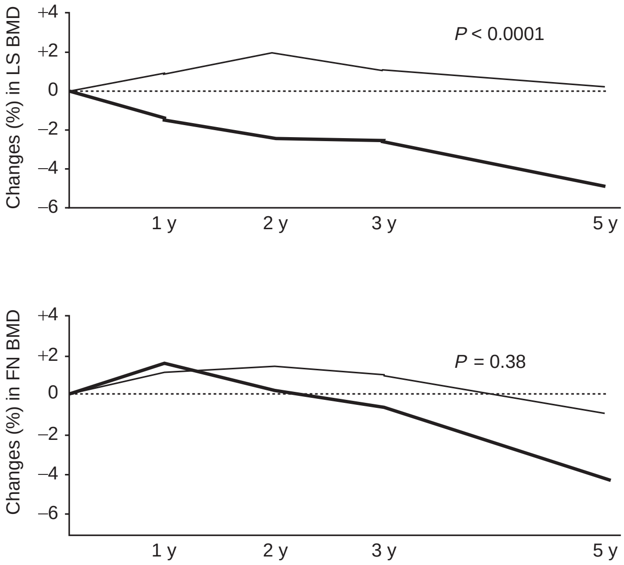

**Figure 1** Age and clodronate adjusted changes from baseline to 5 years in BMD of lumbar spine and femoral neck in HRT non-users (plain line) and HRT recent users (bold line)

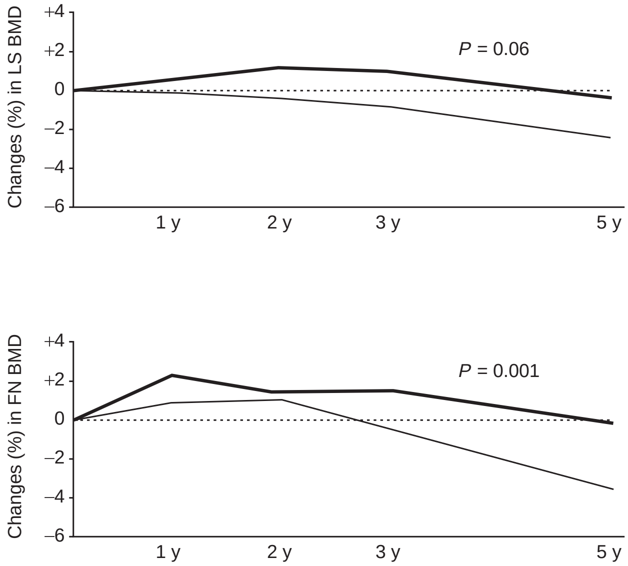

**Figure 2** Age and previous HRT adjusted changes from baseline to 5 years in BMD of lumbar spine and femoral neck in the clodronate (bold line) and the control (plain line) groups

### The effect of previous HRT on BMD

In recent users of HRT there was more significant bone loss in lumbar spine –3.0% (–5.1 to –0.9) than in HRT non-users +1.2% (–0.2 to +2.6) at 3 years ($P$ < 0.0001). No significant differences were found in femoral neck between the groups at 3 years: –0.4% (–3.6 to +2.8) vs. + 1.7% (–0.3 to +3.7), respectively ($P$ = 0.27). At the end of 5 years follow-up, 2 years after finishing all antiresorptive treatment, the changes from baseline were +0.5% (–1.3 to +2.3) in the lumbar spine and –1.5% (–3.6 to +0.6) in the femoral neck in HRT non-user, while in HRT recent users the respective changes were –6.5% (–9.4 to –3.6) and –4.8% (–8.4 to –1.2). The total differences between the HRT non-users and recent users in BMD of lumbar spine and femoral neck at five years were 7.0%

**Table 2** Percentile changes (mean and 95% CI) from baseline in BMD of lumbar spine and femur at 3 years in the clodronate and control groups according to previous HRT

| BMD | HRT non-users | HRT recent users | HRT non-users | HRT recent users |
|---|---|---|---|---|
| | at 3 years | | at 5 years | |
| Lumbar spine | | | | |
| Control | 0.0%<br>(–1.9 to +1.9) | –3.8%<br>(–6.3 to –1.3) | –0.2%<br>(–2.8 to +2.4) | –7.1%<br>(–11.5 to –2.7) |
| Clodronate | +2.3%<br>(+0.4 to +4.2) | –1.8%<br>(–6.3 to +2.7) | +1.0%<br>(–1.6 to +3.6) | –5.5%<br>(–9.8 to –1.2) |
| Femoral neck | | | | |
| Control | +1.2%<br>(–1.8 to +4.2) | –2.3%<br>(–5.4 to +0.8) | –3.0%<br>(–6.6 to +0.6) | –8.1%<br>(–11.5 to –4.7) |
| Clodronate | +2.2%<br>(–0.6 to +5.0) | +2.5%<br>(–4.5 to +9.5) | –0.2%<br>(–2.8 to +2.4) | +0.2%<br>(–6.6 to +7.0) |

© 2001 Cancer Research Campaign and 3.3% in favour of HRT non-users ($P$ < 0.0001 and 0.38, respectively) (Figure 1).

### The effect of clodronate on BMD

During clodronate treatment there was no significant bone loss in lumbar spine +1.0% (–0.9 to +2.9) and femoral neck +2.4% (–0.4 to +5.0) as compared to the controls –1.7% (–3.3 to –0.1) and – 0.4% (–2.5 to +1.7) at 3 years ($P$ = 0.01 and 0.12, respectively). After 5 years from the beginning of the study, 2 years after finishing the treatment, there was still less bone loss in the clodronate than in the control group, especially in femoral neck: lumbar spine –1.0% (–3.4 to +1.4) vs. –3.2% (–5.8 to –0.6), and femoral neck –0.1% (–2.6 to +2.4) vs. –5.2% (–7.7 to –2.7) ($P$ = 0.06 and 0.001, respectively) (Figure 2). This was seen especially in HRT non-users (Table 2).

### The effect of different antioestrogens on BMD

No differences were found in BMD of lumbar spine and femoral neck at 3 years between the tamoxifen and toremifene groups in patients treated with antioestrogen only: bone loss in lumbar spine –2.3% (–4.9 to +0.3) and –1.0% (–3.1 to +1.1) and in femoral neck 0% (–3.8 to +3.8) and –0.7% (–3.2 to +1.8) at 3 years, respectively. However, there were somewhat more HRT recent users in the tamoxifen group than in the toremifene group.

### DISCUSSION

Our results indicate that among postmenopausal women who discontinue HRT because of breast cancer, withdrawal of HRT is associated with more rapid bone loss than in those women who have not used HRT at least within a year before the adjuvant therapy. This was seen especially in the lumbar spine, where the bone loss rate after withdrawal of HRT was more significant than in the femoral neck due to more rapid bone turnover rate in trabecular bone than in cortical bone. Neither antioestrogen therapy alone nor antioestrogen with clodronate could totally prevent the bone loss related to withdrawal of HRT especially in lumbar spine. However, addition of clodronate to the antioestrogen therapy seemed to retard the bone loss rate more than antioestrogen alone.

Despite the limited sample size and the retrospective nature of the study, the study arms were well balanced except for age and weight. Patients who had recently used HRT were significantly younger and weighed less than those women who had not used HRT at least within a year before breast cancer treatment, which may have exaggerated the negative effect of previous HRT on BMD changes. In regression analyses age but not weight had a significant effect on BMD changes. However, after adjusting the data by age and clodronate treatment the previous HRT still had a significant effect on BMD changes especially in lumbar spine.

Oestrogen withdrawal in postmenopausal women is known to induce bone loss. In 2 previous studies of Lindsay et al (1978) and Christiansen et al (1981) the annual bone loss of women with mean age of 47 or 51 years was 2 to 2.5% per year during the first 3 or 4 years after the withdrawal of oestrogen replacement therapy, slowing down within the next 4 years. Tamoxifen has been demonstrated to prevent bone loss in HRT-naive postmenopausal women, but in premenopausal women, on the contrary, it induces a bone loss (Powles et al, 1996). In line with prior investigations of tamoxifen, in the present study in patients who were treated with antioestrogen alone, no bone loss was seen neither in lumbar spine nor in femoral neck during the antioestrogen therapy who had not used HRT within a year before the therapy (Love et al, 1992; Ward et al, 1993; Kristensen et al, 1994; Grey et al, 1995; Powles et al, 1996). No data have previously been available on the bone-maintaining effects of tamoxifen in women who have discontinued HRT. Our reslts show that after HRT the BMD decreased both in lumbar spine and femur despite the antioestrogen therapy. The annual bone loss, however, during the 3-year antioestrogen therapy seemed to be somewhat less in the present study (– 1.3% in lumbar spine and –0.8% in femoral neck per year) than in the previous studies without antioestrogen therapy (2.0–2.5% per year), which might indicate a partially preventive effect of antioestrogen therapy after withdrawal of HRT (Lindsay et al, 1978; Christiansen et al, 1981). Similarly, the annual bone loss rate in the present study during the antioestrogen therapy seems to be at the same level as in postmenopausal women after natural menopause (lumbar spine – 1.24 ± 1.5% and femoral neck – 0.48 ± 0.8%), but less than in perimenopausal women (lumbar spine – 2.35 ± 1.5% and femoral neck – 1.82 ± 1.1%) (Pouilles et al, 1993, 1995).

The present results confirm our previous findings of 2-year antioestrogen therapy with tamoxifen or toremifene (Saarto et al, 1997b), that the novel antioestrogen toremifene shares the partial oestrogen agonistic effect on bone with tamoxifen. No significant differences were found between tamoxifen and toremifene neither in the lumbar spine nor in femoral neck with 3-year treatment. Our results differ from those of Marttunen et al, where tamoxifen was superior to toremifene in prevention of bone loss in 30 postmenopausal women with primary breast cancer (Marttunen et al, 1998). This difference between the studies could be due to chance because of small number of patients, but also due to the lower dose (40 mg) of toremifene in their report.

We have previously demonstrated that 2-year adjuvant clodronate treatment improved BMD of lumbar spine and femoral neck in postmenopausal women treated with adjuvant antioestrogen therapy (Saarto et al, 1997b). At 3 years of the treatment and even 2 years after the withdrawal of clodronate therapy BMD was still better preserved in the clodronate group as compared to antioestrogen alone, even though these differences were not statistically significant due to limited statistical power. However, 3-year clodronate plus antioestrogen therapy could not totally prevent the bone loss related to HRT withdrawal especially in lumbar spine, where the bone loss was more marked. In line with our previous findings in premenopausal breast cancer patients with chemotherapy induced ovarian failure and a rapid bone loss in lumbar spine, clodronate reduced the bone loss only partially, even though the less marked bone loss in menstruating women was totally prevented (Saarto et al, 1997a). Comparable studies are not available to show weather other bisphosphonates could have been more powerful than clodronate to prevent HRT-withdrawal induced bone loss.

Our results indicate that postmenopausal women who have recently discontinued HRT have more rapid bone loss than women who have not used HRT within a year. 3-year antiresorptive therapy with antioestrogen alone or antioestrogen and clodronate could not totally prevent the bone loss related to HRT withdrawal especially in lumbar spine, even though it seemed to retard it.

### ACKNOWLEDGEMENTS

We want to express our gratitude to Professor Seppo Sarna, Ph.D. from Department of Public Health (Biostatistics) University of

 *© 2001 Cancer Research Campaign*

Helsinki, for the advice and valuable contribution to the statistical analyses.

### REFERENCES

Black DM, Cummings SR, Karpf DB, Cayley JA, Thompson DE, Nevitt MC, Bauer DC, Genant HK, Haskel WL, Marcus R, Ott SM, Torner JC, Quandt SA, Reiss TF and Ensrud KE (1996) Randomised trial of effect of alendronate on risk of fracture in women with existing vertebral fractures. *Lancet* **348**: 1535–1541

Christiansen C, Christensen MS and Transbol I (1981) Bone mass in postmenopausal women after withdrawal of oestrogen/gestagen replacement therapy. *Lancet* **1**: 459–461

Cummings SR, Black DM, Thompson DE, Applegate We, Barret-Connor E and Musliner TA (1998) Effect of alendronate on risk of fracture in women with low bone density but without vertebral fractures: results from the fracture intervention trial. *JAMA* **280(24)**: 2077–2082

Fleisch H (1995) *Bisphosphonates in bone disease. From the laboratory to the patient*. The Parthenon Publishing Group: New York, London

Grey AB, Stapleton JP, Evans MC, Tatnell MA, Ames RW and Reid IR (1995) The effect of the antiestrogen tamoxifen on bone mineral density in normal late postmenopausal women. *Am J Med* **99**: 636–641

Harris ST, Watts NB, Jackson RD, Genant HK, Wasnich RD, Ross P, Miller PD, Licata AA and Chesnut III CH (1993) Four-year study of intermittent cyclic etidronate treatment of postmenopausal osteoporosis: three years of blinded therapy followed by one year of open therapy. *Am J Med* **95**: 557–567

Hosking D, Chilvers CED, Christiansen C, Ravn P, Wasnich R and Ross P (1998) Prevention of bone loss with alendronate in postmenopausal women under 60 years of age. *New Engl J Med* **338(8)**: 485–492

Karpf DB, Sharpiro DR, Seeman E, Ensrud KE and Johnston CCJ (1997) Prevention of nonvertebral fractures by alendronate: A meta-analysis. *JAMA* **277(14)**: 1159–1164

Kristensen B, Ejlertsen B, Dalgaard P, Larsen L, Holmegaard SN, Transbol I and Mouridsen HT (1994) Tamoxifen and bone metabolism in postmenopausal low-risk breast cancer patients: a randomized study. *J Clin Oncol* **12**: 992–7

Liberman UR, Weiss SR, Bröll J, Minne HW, Quan H, Bell NH, Rodriguez-Portales J, Downs RW, Dequeker J, Favus M, Seeman E, Recker R, Capizzi T, Santora II AC, Lombardi A, Shah RV, Hirch LJ and Karpf DB (1995) Effect of oral alendronate on bone minenral density and the incidence of fractures in postmenopausal osteoporosis. *N Engl J Med* **333**: 1437–1443

Lindsay R, Hart DM, MacLean A, Clark AC, Kraszewski A and Garwood J (1978) Bone response to termination of oestrogen treatment. *Lancet* **1**: 1325–1327

Love RR, Mazess RB, Barden HS, Epstein S, Newcomb PA, Jordan VC, Carbone PP and DeMets DL (1992) Effects of tamoxifen on bone mineral density in postmenopausal women with breast cancer. *N Engl J Med* **326**: 852–856

Marttunen MB, Hietanen P, Tiitinen A and Ylikorkala O (1998) Comparison of effects of tamoxifen and toremifene on bone biochemistry and bone mineral density in postmenopausal breast cancer patients. *J Clin Endocrinol Metab* **83**: 1158–1162

Pouilles JM, Tremollieres F and Ribot C (1993) The effects of menopause on longitudinal bone loss from the spine. *Calcif Tissue Int* **52**: 340–343

Pouilles JM, Tremollieres F and Ribot C (1995) Effect of menopause on femoral and vertebral bone loss. *J Bone Miner Res* **10**: 1531–1536

Powles TJ, Hickish T, Kanis JA, Tidy A and Ashley S (1996) Effect of tamoxifen on bone mineral density measured by dual-energy X-ray absorptiometry in healthy premenopausal and postmenopausal women. *J Clin Oncol* **14**: 78–84

Saarto T, Blomqvist C, Välimäki M, Sarna S and Elomaa I (1997a). Chemical castration induced by adjuvant CMF therapy causes a rapid bone loss which is reduced by clodronate. A randomized study in premenopausal breast cancer women. *J Clin Oncol* **15**: 1341–1347

Saarto T, Blomqvist C, Välimäki M, Sarna S and Elomaa I (1997b) Clodronate improves bone mineral density in postmenopausal breast cancer women treated with adjuvant antiestrogens. *Br J Cancer* **75**: 602–605

Storm T, Thamsborg G, Steiniche T, Genant HK and Sorensen OH (1990) Effect of intermittent cyclical etidronate therapy on bone mass and fracture rate in women with postmenopausal osteoporosis. *N Engl J Med* **322**: 1265–1271

Valavaara R, Pyrhönen S, Heikkinen M, Rissanen P, Blanco G, Tholix E, Nordman E, Taskinen P, Holsti L and Hajba A (1988) Toremifene, a new antiestrogenic compound, for treatment of advanced breast cancer. Phase II study. *Eur J Cancer Clin Oncol* **24**: 785–790

Ward RL, Morgan G, Dalley D and Kelly PJ (1993) Tamoxifen reduces bone turnover and prevents lumbar spine and proximal femoral bone loss in early postmenopausal women. *Bone Miner* **22**: 87–94

Watts NB, Harris ST, Genant HK, Wasnich RD, Miller PD, Jackson RD, Licata AA, Ross P, Woodson G3, Yanover MJ, Mysiw WJ, Kohse L, Rao MB, Steiger P, Richmond B and Chesnut III CH (1990) Intermittent cyclical etidronate treatment of postmenopausal osteoporosis. *N Engl J Med* **323**: 73–79

© 2001 Cancer Research Campaign
